# Effects of chronic psychosocial stress on the development of mouse oocytes and preimplantation embryos

**DOI:** 10.18699/vjgb-26-58

**Published:** 2026-07

**Authors:** T.N. Igonina, I.D. Chekashov, A.L. Levinson, S.Ya. Amstislavsky

**Affiliations:** Institute of Cytology and Genetics of the Siberian Branch of the Russian Academy of Sciences, Novosibirsk, Russia; Institute of Cytology and Genetics of the Siberian Branch of the Russian Academy of Sciences, Novosibirsk, Russia Novosibirsk State University, Novosibirsk, Russia; Novosibirsk State University, Novosibirsk, Russia; Institute of Cytology and Genetics of the Siberian Branch of the Russian Academy of Sciences, Novosibirsk, Russia

**Keywords:** chronic psychosocial stress, oocytes, embryos, in vitro fertilization, early embryonic development, TUNEL assay, apoptosis, assisted reproductive technologies (ART), хронический психосоциальный стресс, ооциты, эмбрионы, оплодотворение in vitro, раннее эмбриональное развитие, TUNEL-анализ, апоптоз, вспомогательные репродуктивные технологии (ВРТ)

## Abstract

The impact of psychosocial chronic stress on mammalian oocyte maturation, fertilization and early stages of embryonic development remains poorly understood. This study addresses the effects of chronic psychosocial stress on the reproductive outcome in female mice, i. e. the development of in vitro- and in vivo-derived embryos. The model of chronic stress used in the study comprised a 21-day protocol consisting of a period of social isolation followed by the overcrowding. Two experiments were conducted, varying in the type of fertilization. In experiment 1, female mice were stressed at the folliculogenesis stages; then oocyte maturation and in vitro fertilization were performed, and the early development of the in vitro-derived embryos was studied. Experiment 2 differed in that fertilization was performed in vivo, and the resulting in vivo-derived embryos were cultured in vitro since the two-cell stage. To assess preimplantation embryo development, blastocysts were fixed, stained with the TUNEL/DAPI method and analyzed using fluorescence microscopy, i. e. the number of interphase nuclei and the apoptosis index were estimated. The results of Experiment 1 showed that chronic stress did not affect oocyte maturation or their fertilization capacity. However, embryos from the stress group contained fewer interphase nuclei (p < 0.001), which points to a lower cleavage rate. Meanwhile, the apoptosis rate in these blastocysts was comparable to controls. Experiment 2 showed that chronic stress caused a decrease in the proportion of embryos that achieved the blastocyst stage during the culture period and an increase in the proportion of morulae (p < 0.01), as well as a decrease in the number of interphase nuclei in blastocysts (p < 0.001). Experiments demonstrated that the chronic psychosocial stress exerts a moderate but significant effect on early embryonic development, primarily via the reduced proliferative activity of embryonic cells. These results obtained in mice have a translational value for reproductive medicine and highlight the importance of maternal stress when analyzing ART outcomes.

## Abstract (ru)

Эффекты хронического психосоциального стресса на созревание ооцитов, оплодотворение и ранние этапы развития эмбрионов млекопитающих остаются недостаточно изученными. Настоящее исследование направлено на изучение влияния хронического психосоциального стресса на репродуктивные показатели самок мышей и развитие эмбрионов, полученных после оплодотворения in vitro и in vivo. Хронический стресс моделировали с помощью 21-дневного протокола, включавшего период социальной изоляции, за которым следовало содержание в условиях высокой скученности. Проведены два эксперимента c воздействием стресса на стадии фолликулогенеза, различавшихся способом оплодотворения. В эксперименте 1 у самок мышей после воздействия стресса были извлечены ооциты, проведено дозревание ооцитов in vitro, экстракорпоральное оплодотворение и исследовано раннее развитие полученных эмбрионов. Эксперимент 2 отличался тем, что оплодотворение было in vivo, полученные эмбрионы культивировали in vitro с двухклеточной стадии. Для оценки преимплантационного развития эмбрионов бластоцисты фиксировали, окрашивали методом TUNEL/DAPI и анализировали с помощью флуоресцентной микроскопии, подсчитывая число интерфазных ядер и индекс апоптоза. Эксперимент 1 показал, что хронический стресс не оказывает влияния на дозревание ооцитов и их способность к оплодотворению. Однако эмбрионы из группы «стресс» содержали меньшее число интерфазных ядер (p < 0.001), что указывает на замедленную скорость дробления. Между тем уровень апоптоза в этих бластоцистах был сопоставим с таковым в контрольной группе. В эксперименте 2 хронический стресс вызвал снижение процентной доли эмбрионов, достигших стадии бластоцисты в течение периода культивирования, и увеличение доли морул (p < 0.01), а также снижение числа интерфазных ядер в бластоцистах (p < 0.001). Эксперименты показали, что хронический психосоциальный стресс оказывает умеренное, но значимое влияние на раннее эмбриональное развитие, главным образом за счет снижения пролиферативной активности клеток. Эти результаты, полученные на мышах, имеют трансляционное значение для репродуктивной медицины и подчеркивают важность учета материнского стресса при анализе исходов ВРТ.

## Introduction

Chronic psychosocial stress has become an essential feature of life in a modern urban agglomeration. Urbanization is associated with a complex of predominantly psychological stressors, including social overload (crowding, excess of superficial contacts) combined with a deficit of meaningful connections, professional loads, and uncertainty. These stressors adversely affect the psychoemotional state and reproductive function (Ochnik et al., 2024; Orquiza, 2024).

Chronic stress activates the hypothalamic-pituitary-adrenal (HPA) axis, resulting in an increase in glucocorticoid synthesis (James et al., 2023). This affects the reproductive function through direct and indirect mechanisms (Zhai et al., 2020; Bhaumik et al., 2023; Jeon et al., 2023).

The direct effects are mediated by glucocorticoid receptors present in ovarian cells (granulosa, theca cells) and in the early embryo (Bhaumik et al., 2023; Jeon et al., 2023). In the ovary, this leads to the suppression of proliferation and induction of apoptosis of granulosa cells, disruption of steroidogenesis, and deterioration of oocyte quality (Prasad et al., 2016; Bhaumik et al., 2023). At the preimplantation stage of embryo development, elevated maternal corticosterone levels can directly suppress embryonic cell division, reduce the number of cells in the blastocyst, and lead to delayed hatching (Liu et al., 2012; Zhai et al., 2020).

An indirect mechanism of stress influence on reproduction is the suppression of the hypothalamic-pituitary-gonadal (HPG) axis. High glucocorticoid levels inhibit gonadotropin-releasing hormone (GnRH) secretion in the hypothalamus, which leads to a decrease in the production of gonadotropins, i. e. luteinizing (LH) and follicle-stimulating (FSH) hormones (Zhou et al., 2019). A lack of these hormones disrupts key ovarian processes: folliculogenesis, ovulation, and steroid synthesis (Zhai et al., 2020).

Such complex dysregulation causes risks for reproductive health and can contribute to the development of infertility, the prevalence of which reaches 12.6–17.5 % among couples of reproductive age (Cox et al., 2022). Assisted reproductive technologies (ARTs) are increasingly used to treat infertility in clinical practice (Abdullah et al., 2023). However, data are accumulating that chronic stress is a significant factor capable of influencing the effectiveness of ART (Zhou et al., 2019; Levinson et al., 2022). Most research in this area is focused on studying systemic hormonal changes (Valsamakis et al., 2019), while the effect of stress factors on oocyte maturation and early embryo development in the context of ART application remains poorly studied. The significance of such studies nowadays is high as the number of women resorting to ART procedures increases (Chambers et al., 2021).

Experiments on laboratory animals confirm that stressors of various natures disrupt the HPG axis, changing the hormonal profile in female mice; such changes negatively affect the female reproductive system (Zhai et al., 2020). However, there are only few studies that address the effects of psychosocial stress on female reproductive function and on the outcome of ARTs (Levinson et al., 2022).

In the present study, we used the model of chronic psychosocial stress previously adapted by us for female mice (Lebedeva et al., 2024; Igonina et al., 2024a, b). The main focus of the work was the study of oocyte maturation in vitro and in vivo, measured as the effectiveness of IVF, and the subsequent embryo development under chronic psychosocial stress. The novelty of the approach includes modeling chronic psychosocial stress in female mice combining with subsequent assessment of its impact on the key stages of ART protocols, from oocyte maturation to embryo culture, which ensures the high translational value of this work.

The aim of this study is to elucidate the effect of chronic psychosocial stress on the reproductive function in female mice and on early development of in vitro-derived and in vivoderived embryos.

## Materials and methods

Experimental animals. Studies were conducted on sexually mature females (n = 27) and fertile males (n = 25) of the CD1 mouse strain aged 2.5 months, raised in the specific pathogen free (SPF) vivarium of the Institute of Cytology and Genetics SB RAS (ICG SB RAS). Animals were housed in individually ventilated OptiMice cages (Animal Care, United States) measuring 34.3 × 29.2 × 15.5 cm (length × width × height) at a temperature of 22–24 °C and relative humidity of 40–50 %. Sterile fractionated birch chips for laboratory animals (TU 16.10.23-001-0084157135-2019) were used as bedding. All animals had free access to standardized autoclaved Delta Feeds chew for laboratory mice and rats (BioPro, Russia) and purified water “Severyanka” (Ekoproekt, Russia) enriched with mineral supplements. A light regime of 12 h light/12 h dark (lights on at 3:00 am) was maintained throughout the entire experiment period.

Chronic psychosocial stress modeling. Animals of the experimental group were kept under conditions of social isolation (one female per cage) for 11 days; then they were transferred to crowding conditions (social overload): 11 females per cage for the next 10 days. The total duration of the stress protocol was 21 days. Animals in the control group were kept under standard vivarium conditions: groups of 5 females per cage. This stress model was verified by evaluation of corticosterone levels in the blood serum.

Experimental design. Female mice were randomly distributed between two groups: control group – no stress; stress group – chronic psychosocial stress according to the protocol described above.

**Fig. 1.**
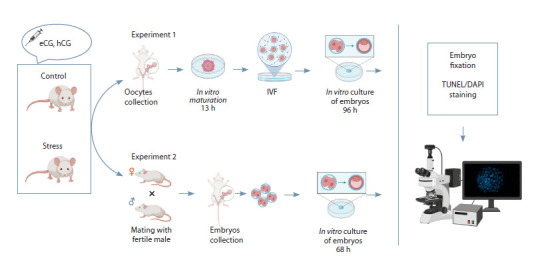
Experimental design. eCG – equine chorionic gonadotropin, hCG – human chorionic gonadotropin.

To evaluate the effect of chronic psychosocial stress on reproductive function and early embryonic development, two sequential experiments were conducted, differing in the method of fertilization (Fig. 1). All the females used in the experiments were hormonally stimulated for superovulation (protocol presented below).

Experiment 1. Effect of stress on oocyte maturation, in vitro fertilization, and early embryo development. Females from both groups (control, n = 6; stress, n = 6) were euthanized four hours after hCG administration by plunging to carbon dioxide (CO2). Blood and ovaries were collected. Immature oocytes at the germinal vesicle (GV) stage were separated from ovarian tissue. Oocytes were matured in vitro, then fertilized using IVF, and the resulting embryos were cultured in a CO2 incubator for 96 hours. The in vitroderived embryos were then fixed in 4 % formalin dissolved in phosphate-buffered saline, stained with TUNEL/DAPI, and analyzed using fluorescence microscopy.

Experiment 2. Effect of stress on the development of in vivo fertilized embryos. Females from both groups (control, n = 8; stress, n = 7) were mated with fertile males. The animals were euthanized 48 hours after pairing using carbon dioxide, and blood was collected. Embryos were collected from the oviducts at the stage of 2–4-cells. The collected embryos were cultured in vitro in a CO2 incubator for 68 hours. After culturing, the embryos were fixed, stained with TUNEL/DAPI, and analyzed using fluorescence microscopy

Corticosterone assay in blood serum. Blood (0.5 ml) was collected into tubes immediately after decapitation of the animals. Samples were incubated for 25 min at room temperature for clot formation and centrifuged (1,000g, 10 min; Hermle Z 326, Germany). The resulting serum was stored at –80 °C. Serum corticosterone concentration was determined by enzyme-linked immunosorbent assay using the Corticosterone rat/mouse–IFA kit (Chema, Russia) according to manufacturer’s recommendations. Optical density was measured at 450 nm on a plate spectrophotometer (Thermo Fisher Scientific, United States) 15 min after stopping the reaction. The concentration was calculated using a calibration curve.

Hormonal stimulation of ovaries. For superovulation, mice were injected intraperitoneally with 7.5 IU of equine chorionic gonadotropin (eCG, folligon, Intervet, Netherlands), and after 46 hours, 7.5 IU of human chorionic gonadotropin (hCG, chorulon, Intervet, Netherlands) in accordance with the standard superovulation protocol accepted for mice (Shindo et al., 2022).

Immature oocyte collection and in vitro maturation. Euthanasia was performed 4 hours after hCG injection. Ovaries were removed from the surrounding tissues and placed into pre-warmed FertiCult Flushing medium (FertiPro, Belgium). Immature oocytes at the germinal vesicle (GV) stage were carefully separated from ovarian tissue using a thin needle and tweezers. Oocytes were transferred onto Petri dishes (Corning Incorporated, United States), placed into 50 μL drops of HTF medium pre-equilibrated in a CO2 incubator, and covered with sterile mineral oil. The culturing was carried out in a CO2 incubator (New Brunswick™ Galaxy 48R, Eppendorf, Germany) at 37 °C, 5 % CO2, and 90 % humidity for 13–14 hours. Oocyte maturity was assessed by the presence of the first polar body; matured oocytes were then used for IVF.

Collection of epididymal sperm. Epididymides were taken from euthanized males. The tissue was placed in 100 μL of prewarmed HTF medium onto Petri dishes (Corning Incorporated, United States) and cut to allow sperm cells to flow out. The dishes with sperm cells were incubated for 30 minutes at 37 °C and 5 % CO2 aiming at spermatozoa capacitation. Then 10 μL of the suspension was transferred to 60 μL of fresh HTF medium and incubated for another 30 minutes. The resulting material was used for IVF.

In vitro fertilization. Mature oocytes were incubated for 30 minutes in 60 μL drops of pre-equilibrated HTF medium under mineral oil (FertiPro, Belgium). Then 10 μL of epididymal sperm suspension was added, and the dishes with medium drops containing oocytes and sperm cells were placed in a CO2 incubator for 4 hours. After incubation, oocytes were washed and transferred to KSOM AA medium for further culturing. Successful fertilization was confirmed by the presence of two pronuclei in the oocytes.

Collection of in vivo-derived embryos. Immediately after hCG injection, females were mated with fertile males. Mating success was determined after 18 hours by the presence of a copulatory plug and/or spermatozoa in vaginal smears. The fertilized mice were euthanized 48 hours after hCG injection. Then the oviducts and uterus were removed and flushed with FertiCult Flushing medium solution (FertiPro, Belgium) to obtain embryos. Embryos at the 2–4 blastomere stage were collected for in vitro cultivation.In vitro embryo culture. Embryos were cultured in KSOM AA medium in 20 μL drops under mineral oil in a CO2 incubator (New Brunswick™ Galaxy 48R, Eppendorf, Germany) at 37 °C, 5 % CO2, and 90 % humidity.The embryo quality and the rate of embryo development were assessed daily using light microscopy (Leica S80 APO stereomicroscope, Germany). For embryos at cleavage stages, the number, shape, and size of blastomere, as well as their general morphology, were analyzed. For blastocysts, blastocoel size, inner cell mass (ICM) compactness, and trophectoderm (TE) cell density were assessed. An embryo was considered normally developing if it corresponded to the expected stage after the culture period and had no signs of damage (vacuolization, cytoplasm granulation), the degree of fragmentation did not exceed 20 %, blastocysts had a compact ICM, and the blastocoel was surrounded by TE cells. If there was no cell number increase in a particular embryo or the transition to the next developmental stage within 24 hours of observation not happened within 24 hours of in vitro culturing, embryos were classified as non-developing. In addition to the qualitative assessments (developing/non-developing), the percentage of embryos that reached each specific stage was used in the subsequent statistical analysis of in vitro development dynamics.DAPI and TUNEL staining of blastocysts followed by fluorescence microscopy. Embryos were fixed and stained using the TUNEL FITC kit (Vazyme, China) according to the manufacturer’s protocol. After three washes with phosphatebuffered saline, samples were treated with proteinase K (5 min), incubated in equilibration buffer (15 min), and then in TUNEL solution (1 hour, 37 °C). Thereafter, the embryos were washed, stained with DAPI (5 min), and mounted on slides. Microscopic evaluation was performed with a Zeiss Axio Imager 2 microscope (Germany) using DAPI-FITC filters. Nuclei and apoptotic cells were counted using ImageJ software. The apoptosis index was calculated as the ratio of apoptotic cells to the total number of cells.

We analyzed 124 randomly selected embryos obtained in vitro by IVF (65 in the control group and 59 in the stress group) and 274 embryos obtained in vivo after natural mating (166 in the control group and 108 in the stress group).

Preparation of culture media. Human tubal fluid (HTF) medium (Quinn et al., 1985) was prepared for IVF. A modification of this medium (HTFm) was used for oocyte maturation. Embryos were cultured in KSOM AA according to (Chatot et al., 1989). All media were prepared independently from individual components (Table S1)1 in purified water, sterilized by filtration through a 0.22-μm membrane, and equilibrated in a CO2 incubator under a layer of mineral oil at 37 °C for ≥4 h.

Supplementary Materials are available in the online version of the paper: https://vavilov.elpub.ru/jour/manager/files/Suppl_Igonina_Engl_30_4.pdf


Statistical analysis. Data were analyzed using STATISTICA v. 8.0 software package (StatSoft Inc). Normality of distribution was checked by the Shapiro–Wilk test. Parameters with normal distribution of data (corticosterone level, percentage of mature and fertilized oocytes) were compared using Student’s t-test. The dynamics of embryo development were assessed by two-way repeated measures ANOVA. Data are presented as M ± SEM. Parameters with non-normal distribution (number of interphase nuclei, apoptosis index) were analyzed using the Mann–Whitney U-test and presented as median with 25 % and 75 % quartiles. Data on embryo development stages were compared using the χ2 (chi-square) test. The significance level was p < 0.05.

## Results

**Verification of the stress model**

**Fig. 2.**
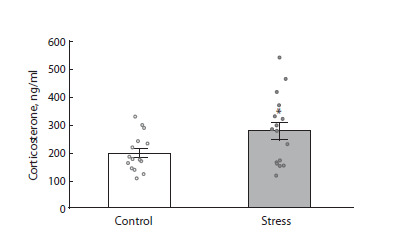
Blood serum corticosterone concentration in control and chronically stressed animals. Data are presented as M ± SEM. Number of animals/samples: n = 15 (control group); n = 16 (stress group). * p < 0.05 compared with the control group (Student’s t-test).

The level of corticosterone in the blood serum in females subjected to chronic psychosocial stress was significantly higher (p < 0.05, Student’s t-test) than in the control group (Fig. 2). This result verifies the stress model used.

**Effect of stress on oocyte maturation, in vitro fertilization, and early embryo development**

**Fig. 3.**
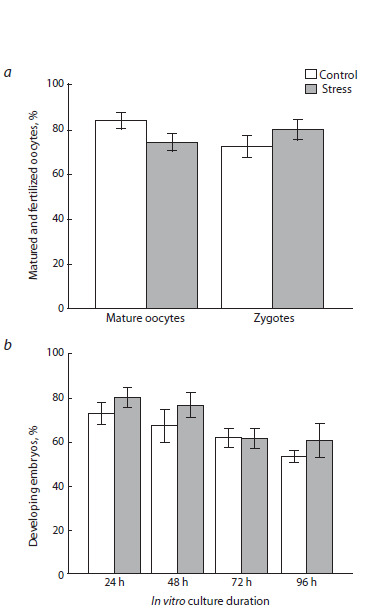
Effect of chronic psychosocial stress on oocyte maturation, their fertilization potential, and in vitro embryo development. a – left: oocytes that reached MII stage (number of mature oocytes in the control group: n = 155, in the stress group: n = 105); right: percentage of zygotes formed after IVF (number of zygotes in the control group: n = 119, in the stress group: n = 88); b – embryo development at different intervals of culturing (initial number of embryos placed in culture in the control group: n = 119, in the stress group: n = 88). Data are presented as M ± SEM (average per female).

From females of the control group (n = 6) and the group subjected to chronic stress (n = 6), 184 and 140 immature oocytes at the germinal vesicle stage (GV) were obtained, respectively. After in vitro culture for 13–14 hours, 155 (84.2 %) oocytes in the control group and 105 (75.0 %) in the stress group matured to the MII stage. Their subsequent fertilization by IVF resulted in zygote formation in 80.4 % (119/155) in the control group and 84.6 % (88/105) in the stress group. Student’s t-test of the obtained data revealed no significant differences between the groups either in the percentage of oocyte maturation (p > 0.05) or in fertilization efficiency (p > 0.05) (Fig. 3a).

Embryo development in vitro proceeded similarly in the control and stress groups at all stages of culturing (Fig. 3b; Table 1). Two-way repeated measures ANOVA revealed no significant effect of chronic psychosocial stress: there was neither a main effect of the “Stress” factor (F(1,10) = 1.2, p >0.05) nor its interaction with the duration of culturing (F(3,30) < 1, p >0.05). Instead, a significant effect of the “Duration of culturing” factor was revealed (F(3,30) = 7.8, p <0.01), reflecting the general dynamics of embryonic development in vitro.

**Table 1.**
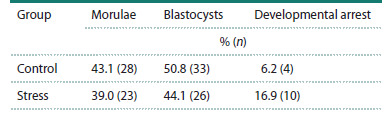
Stages of development of in vitro fertilized embryos after 96 hours of culture

**Fig. 4.**
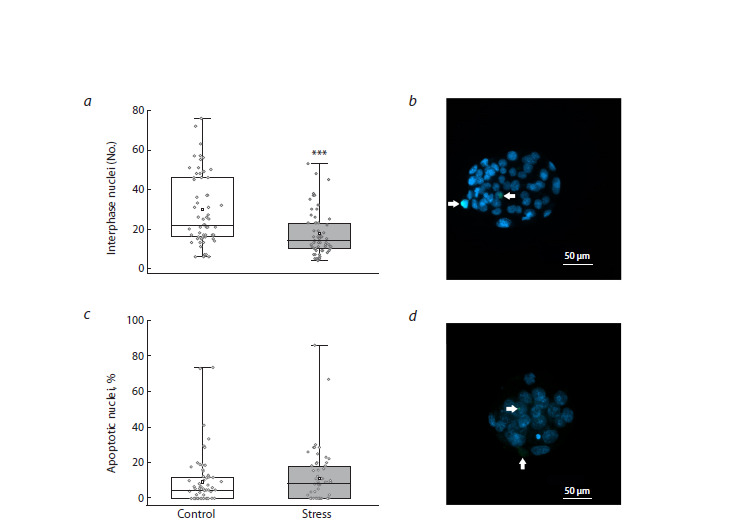
Effect of chronic psychosocial stress on the number of interphase nuclei (a) and the percentage of apoptotic nuclei (c) in mouse embryos fertilized in vitro. Representative images of control (b) and stress (d) group embryos after DAPI (blue, nuclei) and TUNEL (green, apoptotic cells, indicated by arrows) staining. Scale bar 50 μm. Data are presented as median with 25 % and 75 % quartiles. *** p <0.001 (Mann–Whitney U-test). Number of embryos studied: n = 65 in the control group, n = 59 in the stress group.

Embryos obtained by IVF from the stressed females, after 96 hours of in vitro culture, were characterized by a significantly lower number of interphase nuclei compared with the control group (p < 0.001, Mann–Whitney U-test; Fig. 4a). The proportions of apoptotic nuclei did not differ significantly (Fig. 4b).

**Effect of stress on the development of in vivo fertilized embryos**

From the control group females (n = 8) and the females subjected to chronic stress (n = 7), 139 and 115 embryos were obtained, respectively. In the control group, 127 (91.4 %) embryos were at the 2-cell stage, and 12 (8.6 %) were at the 3–4-cell stage; in the stress group, 96 (83.5 %) and 19 (16.5 %) embryos were at the 2-cell and 3–4-cell stages, respectively. Chronic psychosocial stress did not affect the fertility of female mice, as there was no difference between the control (17.4 ± 2.3) and stressed (16.4 ± 1.8) females (p > 0.05, Student’s t-test) in the average number of embryos per female on day 2 of in vivo development.

The rates of the subsequent in vitro embryo development were comparable in the control and stress groups throughout the entire culture period (Fig. S2). Repeated measures ANOVA revealed no statistically significant effect of the “Stress” factor (F(1,13) = 1.20; p > 0.05), the “Duration of culturing” factor (F(2,26) = 2.06; p > 0.05), or interaction between these factors (F(2,26) = 2.82; p> 0.05).

At the late stage of in vitro culturing (68 h), shifts in the distribution of embryo development stages were revealed in the group subjected to chronic stress: the proportion of blastocysts was lower, and the proportion of morulae was higher compared with the control group (p < 0.01; Table 2).

**Table 2.**
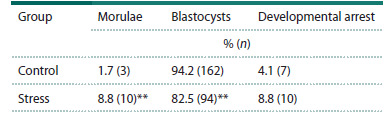
Stages of development of in vivo fertilized embryos after 68 hours of in vitro culture ** p < 0.01 compared with the control group (χ2 test).

**Fig. 5.**
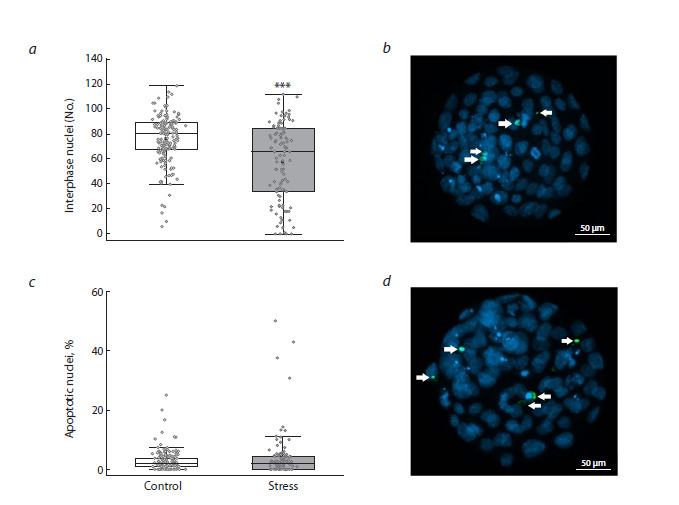
Effect of chronic psychosocial stress on (a) the number of interphase nuclei and (c) the percentage of apoptotic nuclei in in vivo fertilized mouse embryos after 68 hours of in vitro culture. Representative images of (b) control and (d) stress group embryos after DAPI (blue, nuclei) and TUNEL (green, apoptotic cells, indicated by arrows) staining. Scale bar 50 μm. Data are presented as median with 25 % and 75 % quartiles. *** p < 0.001 (Mann–Whitney U-test). Numbers of embryos studied: n = 166 in the control group, n = 108 in the stress group.

Subsequent analysis of embryos cultured in vitro for 68 h revealed a significant decrease in the number of interphase nuclei in the stress group compared with the control group (p < 0.001, Mann–Whitney U-test; Fig. 5a). This result points to a decrease in the proliferative activity of embryonic cells under the influence of stress. However, the proportion of apoptotic cells in the embryos did not differ significantly between the groups (p > 0.05, Mann–Whitney U-test), demonstrating the absence of a pronounced effect of stress on the rates of programmed cell death at this stage of development (Fig. 5b).

## Discussion

Hans Selye was one of the first to define stress from a biological point of view as a nonspecific reaction of the body to a threat to homeostasis (Selye, 1950). Stressors, perceived as a real or potential danger, trigger a cascade of neuroendocrine reactions, the activation of the HPA axis in particular (James et al., 2023). Depending on the duration of exposure, stressors can cause acute or chronic stress (Godoy et al., 2018). In addition, stressors are classified by their nature: physiological, which are associated with a threat to physical well-being, and psychological, which are associated with psychoemotional and psychosocial effects (Godoy et al., 2018). Chronic stress is known to lead to various pathologies (Kalisch et al., 2024).

Studies of the effect of chronic psychoemotional stress on the reproductive system often employ models involving mice, such as “chronic unpredictable stress” and “restriction” (Gao Y. et al., 2016; Levinson et al., 2022). These stressors have both psychological and physical effects, and can cause undesirable consequences, including weight loss, adversely affecting the general condition of the animals (Gao Y. et al., 2016; Furman et al., 2022). In some studies, presenting a predator, e. g. a hungry cat, to a mouse, is used as a model of psychoemotional stress, which creates excessive psychogenic impact associated with a potential threat to life (Liu et al., 2012). Meanwhile, the stressful effects that a person faces in a modern megalopolis are not associated with an immediate threat to life and are mostly psychosocial in nature, while physical stressors are nowadays less relevant (Orquiza, 2024).

In a modern urban environment, a person may simultaneously experience a deficit of meaningful social connections and an abundance of superficial or unwanted contacts associated with high population density and limited privacy (Hammoud et al., 2021; Zhang Z. et al., 2023). This combination of factors enhances the experience of loneliness and social stress, which is confirmed by data on the link between urban crowding and increased physiological stress responses (Lederbogen et al., 2011). Alternating periods of social isolation and forced involvement in intensive social interaction can exacerbate the psychoemotional load and may have a negative impact on the psychophysiological state of a person (Hawkley, Cacioppo, 2010; Lederbogen et al., 2011). Animal studies show that increasing population density and associated crowding exert a pronounced effect on reproductive processes (Suvorov, 2021).

In studies performed on laboratory animals, the effects of social isolation and crowding are addressed so far not in combination, but separately (Gavrilov et al., 2022). In the present study, we used a model based on the alternation of periods of isolation and subsequent overcrowding. A model of this sort was initially offered for rats (Gadek-Michalska et al., 2019) and later adapted for female mice in our studies (Lebedeva et al., 2024; Igonina et al., 2024a, b). Unlike traditional stress models, this model seems to be more relevant to the social conditions faced by women in a modern megalopolis (Ochnik et al., 2024; Orquiza, 2024).

Our results showed that alternating periods of isolation and overcrowding are accompanied by an increase in corticosterone levels in the blood of female mice, which confirms the effectiveness of this model for inducing stress. The results of the present study showed that psychosocial stress did not disrupt oocyte maturation in vitro or affect the oocyte ability to be fertilized. We had demonstrated previously that under in vivo conditions, chronic psychosocial stress led to a decrease in oocyte quality and maturity, accumulation of reactive oxygen species (ROS) in such oocytes, and enhanced apoptosis in cumulus cells (Lebedeva et al., 2024; Igonina et al., 2024b). The pronounced effect of stress on oocyte maturation in vivo versus its absence in vitro is probably explained by the fact that culture media lack the physiological microenvironment necessary for the transmission of the stress signal. This microenvironment, including follicle cells, paracrine and endocrine signals, is known to be necessary for normal oocyte maturation process in vivo (Gilchrist et al., 2004; Yeo et al., 2009). We assume that stress primarily disrupts these communication pathways (Huang, Zeng, 2021; Igonina et al., 2024a). As the direct effect of stress on the oocyte appears to be less significant than its indirect effects through follicular cells and hormonal background (Prasad et al., 2016), oocytes can maintain competence in vitro conditions, where systemic influence is minimized, even after exposure of the female to stress.

Animal studies employing various stress models show that chronic stress causes similar disturbances in folliculogenesis, leading to oocyte maturation impairment. Housing animals under crowded conditions led to high levels of corticosterone and angiotensin II, disrupted hormonal balance, induction of granulosa cell apoptosis, and reduced ovarian reserve (Kim, You, 2022). Stress associated with immobilization caused disruption of meiosis and oocyte death, leading to a decrease in ovarian reserve (Jiang et al., 2023) and an increase in the number of abnormal oocytes (Tsuji et al., 2021). In addition, this type of stress led to disruption of meiotic spindle morphology in oocytes (Sun et al., 2018) and reduced their developmental potential both in vitro and in vivo (Zhang S.Y. et al., 2011; Wu X.F. et al., 2015; Zhao et al., 2020). Chronic unpredictable stress had a similar effect, causing a decrease in ovarian reserve (Gao L. et al., 2020), deterioration of quality, and decrease in the number of oocytes (Wu L.M. et al., 2012a, b). In some cases, it led to the state of anovulation (Kala, Nivsarkar, 2016), disrupted granulosa cell proliferation, and accelerated their aging (Sun et al., 2021; Ma et al., 2024).

The primary systemic pathway mediating the effects of chronic stress is activation of the HPA axis and a sustained increase in circulating glucocorticoid levels (James et al., 2023). Until recently, the literature addressing the question whether or not glucocorticoids directly affect oocytes was area of controversy (Bhaumik et al., 2023); however, it has been shown not long ago that mouse oocytes lack glucocorticoid receptors (GR) (Cincotta et al., 2024). Nevertheless, it is well documented that oocyte maturation is sensitive to elevated glucocorticoid levels via indirect effects mediated by follicular somatic cells, i. e. granulosa and cumulus cells, which express GR (Bhaumik et al., 2023; Cincotta et al., 2024). Glucocorticoid action is realized predominantly through the classical genomic pathway involving binding to the cytoplasmic GR, its translocation to the nucleus, and subsequent regulation of transcription, as well as through rapid non-genomic and isoform-specific mechanisms, resulting in a complex and tissue-specific cellular response (Bhaumik et al., 2023).

In addition, activation of the HPA axis suppresses the HPG axis, including effects at the levels of the hypothalamus and pituitary gland (Bhaumik et al., 2023). It leads to reduced synthesis of sex steroids and disruption of the hormonal milieu required for normal folliculogenesis (Kalantaridou et al., 2004; Whirledge, Cidlowski, 2013). It is worth noting that hormonal stimulation of superovulation was used in the present study, which may compensate for this deficiency and, thereby, attenuate this pathway of stress influence. However, as demonstrated by our data and by results of some other studies, stress continues to exert a negative impact on oocytes through additional mechanisms, primarily via direct disruption of the local ovarian microenvironment, manifesting itself as the induction of granulosa cell apoptosis and alterations in the secretion of paracrine factors (Prasad et al., 2016; Lebedeva et al., 2024; Igonina et al., 2024a). A key element of this process is the action of glucocorticoids on follicular somatic cells (Bhaumik et al., 2023; Cincotta et al., 2024).

Thus, glucocorticoid signaling alters the metabolism of follicular somatic cells, the secretion of local regulatory factors, and the redox status, which in turn induces changes of the oocyte cytoplasm, including the accumulation of maternal mRNA, protein, and epigenetic enzymes (Wu X.F. et al., 2015; Joseph, Whirledge, 2017; Sun et al., 2018). The stress-induced deficit in metabolic and hormonal support from the follicular epithelium brings about suboptimal conditions for the final stages of oocyte maturation.

It should be noted that, in addition to indirect effects mediated by somatic cells, direct sensitivity of oocytes to certain stress-induced signals and hormones, such as corticotropinreleasing factor, has been demonstrated (Zaidan et al., 2013). However, the role of this pathway appears to be substantially smaller than the complex effects mediated through alterations of the follicular microenvironment.

The results of the present study show that embryos obtained from stressed females are characterized by a reduced number of cells in blastocysts regardless of the fertilization method, and, in the case of in vivo fertilization, also by a delay in reaching the blastocyst stage. The time needed to achieve the blastocyst stage and the number of cells in the blastocyst are key indicators of preimplantation development in mammals (Ajduk, Zernicka-Goetz, 2012). A reduction in cell number, in particular, may point to a slowdown in cleavage rates and, more generally, it may be indicative of a low rate of early embryonic development (Nishizono et al., 2017).

A crucial aspect of our findings is that the reduction in blastocyst cell number was observed regardless of whether embryos were obtained via natural in vivo fertilization or by in vitro fertilization of oocytes retrieved from a stress-altered microenvironment but matured in vitro under controlled conditions. Our data support the previously proposed hypothesis that stress-induced changes in the oocyte transcriptome are manifested in embryos derived from these oocytes (Roth, 2018). It appears likely that stress exposure during the early stages of folliculogenesis leads to persistent functional alterations in oocytes, which are attributable, in particular, to epigenetic reprogramming.

Indeed, the earliest preimplantation stages of mammalian embryonic development critically depend on the pool of maternal mRNAs and proteins accumulated in oocytes, the synthesis of which is completed prior to fertilization (Wang et al., 2019; Kojima et al., 2025). These maternal transcripts provide the molecular basis for zygotic genome activation and support rapid cleavage cycles during the early stages of embryonic development, until the embryo’s own transcriptome begins to govern developmental processes (Wang et al., 2019; Kojima et al., 2025). Stress-induced alterations in the abundance, composition, or functional activity of these molecules can disrupt this delicate balance, thereby adversely affecting reproductive success and subsequent stages of embryo development (Roth, 2018). This mechanism may explain the phenomenon observed in our study as well as in others: a reduction in blastocyst cell number and delayed development in embryos from stressed females with the absence of apparent morphological defects in the oocytes (Liu et al., 2012; Casillas et al., 2021).

The hypothesis that the pre-fertilization influence over the oocyte can program embryonic development appears to be broadly applicable, as it is supported by studies not only in mammals (Roth, 2018) but also in fish (Li et al., 2012). In fish, exposure of oocytes to cortisol prior to fertilization results in persistent changes in the embryonic transcriptome and modulates embryonic growth (Li et al., 2012). Importantly, in mammals, unlike in fish, this mechanism appears to be indirect. Since mouse oocytes do not express classical glucocorticoid receptors (Cincotta et al., 2024), the stress signal mainly disrupts the function of follicular somatic cells, which in turn creates conditions for latent reprogramming of the developing gamete.

Thus, our results demonstrate that chronic psychosocial stress, mediated by activation of the HPA axis and disruption of the ovarian microenvironment, induces functional reprogramming of oocytes. This reprogramming does not affect oocyte morphology but predetermines reduced embryo quality and delayed embryonic development. The identified mechanism provides a biological rationale for clinically observed associations between stress and reduced efficacy of assisted reproductive technologies (ART) (Koumparou et al., 2021; Abdoli et al., 2025; Lu et al., 2025). In contrast to the existing inconsistencies in epidemiological data, which may be related to difficulties in the objective assessment of stress (Zanettoullis et al., 2024; Hu et al., 2025), our study indicates that exposure to chronic stress can generate latent oocyte defects with potential consequences for the successful application of reproductive technologies.

## Conclusion

Our study supports the hypothesis of the negative impact of chronic psychosocial stress on reproductive function and provides an experimental basis for the interpretation of clinical observations. The model adequately reproduces key aspects of stress exposure experienced by residents of modern megalopolises. Investigation of the effects of psychoemotional stress on the ovary (Igonina et al., 2024a) and the consequences of these alterations for oocyte development and early embryogenesis opens avenues for the development of novel strategies to mitigate these changes (Wu L.M. et al., 2012b; Igonina et al., 2024b). Integration of such approaches into clinical practice may enable the development of personalized infertility treatment strategies aimed at reducing the negative impact of chronic stress and improving the outcomes of ART programs.

Ethical standards compliance. All studies were approved by the Bioethics Committee of the Institute of Cytology and Genetics, Siberian Branch of the Russian Academy of Sciences (protocol No. 143 of March 15, 2023) and complied with the requirements of the European Directive 2010/63/EU on the protection of animals used for scientific purposes

## Conflict of interest

The authors declare no conflict of interest.

## References

Abdoli S., Gholami A.H., Masoumi S.Z., Najafi-Vosough R., Azimi M.,
Jenabi E., Aliabadi S., Soltanian A.R., Ghaleiha A., Pilehvari S. The
association of anxiety and perceived stress with in vitro fertilization
outcomes in infertile women: a cross-sectional study. J Hum Reprod
Sci. 2025;18(1):16-22. doi 10.4103/jhrs.jhrs_168_24

Abdullah K.A.L., Atazhanova T., Chavez-Badiola A., Shivhare S.B.
Automation in ART: paving the way for the future of infertility treatment.
Reprod Sci. 2023;30(4):1006-1016. doi 10.1007/s43032-022-
00941-y

Ajduk A., Zernicka-Goetz M. Advances in embryo selection methods.
F1000 Biol Rep. 2012;4:11. doi 10.3410/B4-11

Bhaumik S., Lockett J., Cuffe J., Clifton V.L. Glucocorticoids and their
receptor isoforms: roles in female reproduction, pregnancy, and foetal
development. Biology. 2023;12(8):1104. doi 10.3390/biology
12081104

Casillas F., Betancourt M., Juárez-Rojas L., Ducolomb Y., López A.,
Ávila-Quintero A., Zamora J., Ommati M.M., Retana-Márquez S.
Chronic stress detrimentally affects in vivo maturation in rat oocytes
and oocyte viability at all phases of the estrous cycle. Animals
(Basel).
2021;11(9):2478. doi 10.3390/ani11092478

Chambers G.M., Dyer S., Zegers-Hochschild F., de Mouzon J., Ishihara
O., Banker M., Mansour R., Kupka M.S., Adamson G.D. International
Committee for Monitoring Assisted Reproductive Technologies
world report: assisted reproductive technology, 2014. Hum
Reprod. 2021;36(11):2921-2934. doi 10.1093/humrep/deab198

Chatot C.L., Ziomek C.A., Bavister B.D., Lewis J.L., Torres I. An improved
culture medium supports development of random-bred 1-cell
mouse embryos in vitro. J Reprod Fertil. 1989;86(2):679-688. doi
10.1530/jrf.0.0860679

Cincotta S.A., Richardson N., Foecke M.H., Laird D.J. Differential
susceptibility of male and female germ cells to glucocorticoidmediated
signaling. eLife. 2024;12:RP90164. doi 10.7554/eLife.
90164

Cox C.M., Thoma M.E., Tchangalova N., Mburu G., Bornstein M.J.,
Johnson C.L., Kiarie J. Infertility prevalence and the methods of estimation
from 1990 to 2021: a systematic review and meta-analysis.
Hum Reprod Open. 2022;2022(4):hoac051. doi 10.1093/hropen/
hoac051

Furman O., Tsoory M., Chen A. Differential chronic social stress models
in male and female mice. Eur J Neurosci. 2022;55(9-10):2777-
2793. doi 10.1111/ejn.15481

Gadek-Michalska A., Tadeusz J., Bugajski A., Bugajski J. Chronic isolation
stress affects subsequent crowding stress-induced brain Nitric
Oxide Synthase (NOS) isoforms and Hypothalamic-Pituitary-Adrenal
(HPA) axis responses. Neurotox Res. 2019;36(3):523-539. doi
10.1007/s12640-019-00067-1

Gao L., Zhao F., Zhang Y., Wang W., Cao Q. Diminished ovarian reserve
induced by chronic unpredictable stress in C57BL/6 mice. Gynecol
Endocrinol. 2020;36(1):49-54. doi 10.1080/09513590.2019.1631274

Gao Y., Chen F., Kong Q.Q., Ning S.F., Yuan H.J., Lian H.Y., Luo M.J.,
Tan J.H. Stresses on female mice impair oocyte developmental potential:
effects of stress severity and duration on oocytes at the growing
follicle stage. Reprod Sci. 2016;23(9):1148-1157. doi 10.1177/
1933719116630416

Gavrilov V.V., Onufriev M.V., Moiseeva Y.V., Alexandrov Y.I., Gulyaeva
N.V. Chronic social isolation stress and crowding in rats
have different effects on learning an operant behavior and the state
of the hypothalamo-hypophyseal-adrenocortical system. Neurosci
Behav Physi. 2022;52(5):698-704. doi 10.1007/s11055-022-
01295-3

Gilchrist R.B., Ritter L.J., Armstrong D.T. Oocyte-somatic cell interactions
during follicle development in mammals. Anim Reprod Sci.
2004;82-83:431-446. doi 10.1016/j.anireprosci.2004.05.017Godoy L.D., Rossignoli M.T., Delfino-Pereira P., Garcia-Cairasco N.,
de Lima Umeoka E.H. A comprehensive overview on stress neurobiology:
basic concepts and clinical implications. Front Behav
Neurosci.
2018;12:127. doi 10.3389/fnbeh.2018.00127

Hammoud R., Tognin S., Bakolis I., Ivanova D., Fitzpatrick N., Burgess
L., Smythe M., Gibbons J., Davidson N., Mechelli A. Lonely in a
crowd: investigating the association between overcrowding and loneliness
using smartphone technologies. Sci Rep. 2021;11(1):24134.
doi 10.1038/s41598-021-03398-2

Hawkley L.C., Cacioppo J.T. Loneliness matters: a theoretical and empirical
review of consequences and mechanisms. Ann Behav Med.
2010;40(2):218-227. doi 10.1007/s12160-010-9210-8

Hu Y., Wang W., Ma W., Wang W., Ren W., Wang S., Fu F., Li Y. Impact
of psychological stress on ovarian function: insights, mechanisms
and intervention strategies (Review). Int J Mol Med. 2025;55(2):34.
doi 10.3892/ijmm.2024.5475

Huang J., Zeng H. The influence of environmental factors on ovarian
function, follicular genesis, and oocyte quality. Adv Exp Med Biol.
2021;1300:41-62. doi 10.1007/978-981-33-4187-6_3

Igonina T., Lebedeva D., Tsybko A., Rozhkova I., Babochkina T.,
Levinson A., Amstislavsky S. Chronic psychosocial stress affects insulin-
like growth factor 1 and its receptors in mouse ovaries. Reprod
Fertil Dev. 2024a;36:RD24101. doi 10.1071/RD24101

Igonina T.N., Lebedeva D.A., Shavshaeva N.A., Brusentsev E.Yu.,
Levinson A., Amstislavsky S. Effects of in vivo administration of
insulin-like growth factor-1 on oocyte and embryo development
in mice under chronic psychosocial stress. J Evol Biochem Phys.
2024b;60:1725-1740. doi 10.1134/S0022093024050065

James K.A., Stromin J.I., Steenkamp N., Combrinck M.I. Understanding
the relationships between physiological and psychosocial stress,
cortisol and cognition. Front Endocrinol. 2023;14:1085950. doi
10.3389/fendo.2023.1085950

Jeon H., Choi Y., Brännström M., Akin J.W., Curry T.E., Jo M. Cortisol/
glucocorticoid receptor: a critical mediator of the ovulatory process
and luteinization in human periovulatory follicles. Hum Reprod.
2023;38(4):671-685. doi 10.1093/humrep/dead017

Jiang Y., Xu J., Tao C., Lin Y., Lin X., Li K., Liu Q., … Sun Y., Zhang F.,
Kang Y., Xu C., Zhang L. Chronic stress induces meiotic arrest
failure
and ovarian reserve decline via the cAMP signaling pathway.
Front Endocrinol (Lausanne). 2023;14:1177061. doi 10.3389/
fendo.2023.1177061

Joseph D.N., Whirledge S. Stress and the HPA axis: balancing homeostasis
and fertility. Int J Mol Sci. 2017;18(10):2224. doi 10.3390/ijms
18102224

Kala M., Nivsarkar M. Role of cortisol and superoxide dismutase in
psychological stress induced anovulation. Gen Comp Endocrinol.
2016;225:117-124. doi 10.1016/j.ygcen.2015.09.010

Kalantaridou S.N., Makrigiannakis A., Zoumakis E., Chrousos G.P.
Reproductive functions of corticotropin-releasing hormone. Research
and potential clinical utility of antalarmins (CRH receptor
type 1 antagonists). Am J Reprod Immunol. 2004;51(4):269-274. doi
10.1111/j.1600-0897.2004.00155.x

Kalisch R., Russo S.J., Müller M.B. Neurobiology and systems biology
of stress resilience. Physiol Rev. 2024;104(3):1205-1263. doi
10.1152/physrev.00042.2023

Kim J., You S. High housing density-induced chronic stress diminishes
ovarian reserve via granulosa cell apoptosis by angiotensin II overexpression
in mice. Int J Mol Sci. 2022;23(15):8614. doi 10.3390/
ijms23158614

Kojima M.L., Hoppe C., Giraldez A.J. The maternal-to-zygotic transition:
reprogramming of the cytoplasm and nucleus. Nat Rev Genet.
2025;26(4):245-267. doi 10.1038/s41576-024-00792-0

Koumparou M., Bakas P., Pantos K., Economou M., Chrousos G. Stress
management and In Vitro Fertilization (IVF): a pilot randomized
controlled trial. Psychiatriki. 2021;32(4):290-299. doi 10.22365/
jpsych.2021.029

Lebedeva D.A., Igonina T.N., Brusentsev E.Yu., Shavshaeva N.A.,
Amstislavskij
S.Ya. Effects of ovarian stimulation with gonadotropins
in the conditions of chronic psychosocial stress on the quality
of murine oocyte. Russian Journal of Physiology. 2024;110(6):930-
944. doi 10.31857/S0869813924060044 (in Russian)

Lederbogen F., Kirsch P., Haddad L., Streit F., Tost H., Schuch P.,
Wüst S., Pruessner J.C., Rietschel M., Deuschle M., Meyer-Lindenberg
A. City living and urban upbringing affect neural social
stress processing in humans. Nature. 2011;474(7352):498-501. doi
10.1038/nature10190

Levinson A.L., Igonina T.N., Rozhkova I.N., Brusentsev E.Yu., Amstislavskij
S.Ya. Psycho-emotional stress, folliculogenesis, and reproductive
technologies: clinical and experimental data. Vavilovskii
Zhurnal
Genetiki i Selektsii = Vavilov J Genet Breed. 2022;26(5):
431-441. doi 10.18699/VJGB-22-53

Li M., Leatherland J.F., Vijayan M.M., King W.A., Madan P. Glucocorticoid
receptor activation following elevated oocyte cortisol
content is associated with zygote activation, early embryo cell division,
and IGF system gene responses in rainbow trout. J Endocrinol.
2012;215(1):137-149. doi 10.1530/JOE-12-0030

Liu Y.X., Cheng Y.N., Miao Y.L., Wei D.L., Zhao L.H., Luo M.J.,
Tan J.H. Psychological stress on female mice diminishes the developmental
potential of oocytes: a study using the predatory stress
model. PLoS One. 2012;7(10):e48083. doi 10.1371/journal.pone.
0048083

Lu Q., Cheng Y., Zhou Z., Fan J., Chen J., Yan C., Zeng X., Yang J.,
Wang X. Effects of emotions on IVF/ICSI outcomes in infertile
women: a systematic review and meta-analysis. J Assist Reprod
Genet. 2025;42(4):1083-1099. doi 10.1007/s10815-025-03388-7

Ma J., Wang L., Yang D., Luo J., Gao J., Wang J., Guo H., Li J., Wang F.,
Wu J., Hu R. Chronic stress causes ovarian fibrosis to impair female
fertility in mice. Cell Signal. 2024;122:111334. doi 10.1016/j.cellsig.
2024.111334

Nishizono H., Uno K., Abe H. Cleavage speed and blastomere number
in DBA/2J compared with C57BL/6J mouse embryos. J Am Assoc
Lab Anim Sci. 2017;56(1):11-17

Ochnik D., Buława B., Nagel P., Gachowski M., Budziński M. Urbanization,
loneliness and mental health model – a cross-sectional
network analysis with a representative sample. Sci Rep. 2024;14(1):
24974. doi 10.1038/s41598-024-76813-z

Orquiza J.C. Self-stress: a new perspective on stress and moral disorders
of civilization. J Org Psychol. 2024;24(1). doi 10.33423/jop.
v24i1.6885

Prasad S., Tiwari M., Pandey A.N., Shrivastav T.G., Chaube S.K. Impact
of stress on oocyte quality and reproductive outcome. J Biomed
Sci. 2016;23:36. doi 10.1186/s12929-016-0253-4

Quinn P., Kerin J.F., Warnes G.M. Improved pregnancy rate in human
in vitro fertilization with the use of a medium based on the composition
of human tubal fluid. Fertil Steril. 1985;44(4):493-498. doi
10.1016/s0015-0282(16)48918-1

Roth Z. Stress-induced alterations in oocyte transcripts are further expressed
in the developing blastocyst. Mol Reprod Dev. 2018;85(11):
821-835. doi 10.1002/mrd.23045

Selye H. Stress and the general adaptation syndrome. Br Med J. 1950;
1(4667):1383-1392. doi 10.1136/bmj.1.4667.1383

Shindo M., Miyado K., Kang W., Fukami M., Miyado M. Efficient superovulation
and egg collection from mice. Bio Protoc. 2022;12(11):
e4439. doi 10.21769/BioProtoc.4439

Sun J., Guo Y., Zhang Q., Bu S., Li B., Wang Q., Lai D. Chronic restraint
stress disturbs meiotic resumption through APC/C-mediated
cyclin B1 excessive degradation in mouse oocytes. Cell Cycle. 2018;
17(13):1591-1601. doi 10.1080/15384101.2018.1471316

Sun J., Guo Y., Fan Y., Wang Q., Zhang Q., Lai D. Decreased expression
of IDH1 by chronic unpredictable stress suppresses proliferation
and accelerates senescence of granulosa cells through ROS activated
MAPK signaling pathways. Free Radic Biol Med. 2021;169:122-
136. doi 10.1016/j.freeradbiomed.2021.04.016

Suvorov A. Population numbers and reproductive health. Endocrinology.
2021;162(11):bqab154. doi 10.1210/endocr/bqab154

Tsuji A., Ikeda Y., Murakami M., Kitagishi Y., Matsuda S. d-Leucine
protects oocytes from chronic psychological stress in mice. Reprod
Med Biol. 2021;20(4):477-484. doi 10.1002/rmb2.12396

Valsamakis G., Chrousos G., Mastorakos G. Stress, female reproduction
and pregnancy. Psychoneuroendocrinology. 2019;100:48-57.
doi 10.1016/j.psyneuen.2018.09.031

Wang Y., Liu Q., Tang F., Yan L., Qiao J. Epigenetic regulation and
risk factors during the development of human gametes and early embryos.
Annu Rev Genomics Hum Genet. 2019;20:21-40. doi 10.1146/
annurev-genom-083118-015143

Whirledge S., Cidlowski J.A. A role for glucocorticoids in stressimpaired
reproduction: beyond the hypothalamus and pituitary.
Endocrinology. 2013;154(12):4450-4468. doi 10.1210/en.2013-
1652

Wu L.M., Hu M.H., Tong X.H., Han H., Shen N., Jin R.T., Wang W.,
Zhou G.X., He G.P., Liu Y.S. Chronic unpredictable stress decreases
expression of brain-derived neurotrophic factor (BDNF)
in mouse ovaries: relationship to oocytes developmental potential.
PLoS One. 2012a;7(12):e52331. doi 10.1371/journal.pone.
0052331

Wu L.M., Liu Y.S., Tong X.H., Shen N., Jin R.T., Han H., Hu M.H.,
Wang W., Zhou G.X. Inhibition of follicular development induced
by chronic unpredictable stress is associated with growth and differentiation
factor 9 and gonadotropin in mice. Biol Reprod. 2012b;
86(4):121. doi 10.1095/biolreprod.111.093468

Wu X.F., Yuan H.J., Li H., Gong S., Lin J., Miao Y.L., Wang T.Y.,
Tan J.H. Restraint stress on female mice diminishes the developmental
potential of oocytes: roles of chromatin configuration and
histone modification in germinal vesicle stage oocytes. Biol Reprod.
2015;92(1):13. doi 10.1095/biolreprod.114.124396

Yeo C.X., Gilchrist R.B., Lane M. Disruption of bidirectional
oocyte-
cumulus paracrine signaling during in vitro maturation
reduces subsequent mouse oocyte developmental competence.
Biol Reprod. 2009;80(5):1072-1080. doi 10.1095/biolreprod.108.
073908

Zaidan H., Leshem M., Gaisler-Salomon I. Prereproductive stress to
female rats alters corticotropin releasing factor type 1 expression
in ova and behavior and brain corticotropin releasing factor type 1
expression in offspring. Biol Psychiatry. 2013;74(9):680-687. doi
10.1016/j.biopsych.2013.04.014

Zanettoullis A.T., Mastorakos G., Vakas P., Vlahos N., Valsamakis G.
Effect of stress on each of the stages of the IVF procedure: a systematic
review. Int J Mol Sci. 2024;25(2):726. doi 10.3390/ijms
25020726

Zhai Q.Y., Wang J.J., Tian Y., Liu X., Song Z. Review of psychological
stress on oocyte and early embryonic development in female mice.
Reprod Biol Endocrinol. 2020;18(1):101. doi 10.1186/s12958-020-
00657-1

